# The Proteome of Native Adult Müller Glial Cells From Murine Retina*[Fn FN2]

**DOI:** 10.1074/mcp.M115.052183

**Published:** 2015-08-31

**Authors:** Antje Grosche, Alexandra Hauser, Marlen Franziska Lepper, Rebecca Mayo, Christine von Toerne, Juliane Merl-Pham, Stefanie M. Hauck

**Affiliations:** From the ‡Insitute of Human Genetics, University of Regensburg, D-93053 Regensburg, Germany;; §Research Unit Protein Science, Helmholtz Zentrum München, German Research Center for Environmental Health (GmbH), D-85764 Neuherberg, Germany

## Abstract

To date, the proteomic profiling of Müller cells, the dominant macroglia of the retina, has been hampered because of the absence of suitable enrichment methods. We established a novel protocol to isolate native, intact Müller cells from adult murine retinae at excellent purity which retain *in situ* morphology and are well suited for proteomic analyses. Two different strategies of sample preparation - an in StageTips (iST) and a subcellular fractionation approach including cell surface protein profiling were used for quantitative liquid chromatography-mass spectrometry (LC-MSMS) comparing Müller cell-enriched to depleted neuronal fractions. Pathway enrichment analyses on both data sets enabled us to identify Müller cell-specific functions which included focal adhesion kinase signaling, signal transduction mediated by calcium as second messenger, transmembrane neurotransmitter transport and antioxidant activity. Pathways associated with RNA processing, cellular respiration and phototransduction were enriched in the neuronal subpopulation. Proteomic results were validated for selected Müller cell genes by quantitative real time PCR, confirming the high expression levels of numerous members of the angiogenic and anti-inflammatory annexins and antioxidant enzymes (*e.g.* paraoxonase 2, peroxiredoxin 1, 4 and 6). Finally, the significant enrichment of antioxidant proteins in Müller cells was confirmed by measurements on vital retinal cells using the oxidative stress indicator CM-H2DCFDA. In contrast to photoreceptors or bipolar cells, Müller cells were most efficiently protected against H_2_O_2_-induced reactive oxygen species formation, which is in line with the protein repertoire identified in the proteomic profiling. Our novel approach to isolate intact glial cells from adult retina in combination with proteomic profiling enabled the identification of novel Müller glia specific proteins, which were validated as markers and for their functional impact in glial physiology. This provides the basis to allow the discovery of novel glial specializations and will enable us to elucidate the role of Müller cells in retinal pathologies — a topic still controversially discussed.

For many years, research on retinal diseases mainly concentrated on investigations of functional deficits of retinal neurons. Müller cells, the dominant macroglia cells of the retina, were considered passive bystanders. However, owing to their distinct morphology spanning the whole thickness of the retina and being in contact with virtually all retinal cell types enables them to fulfil a plethora of functions which are absolutely essential for neuronal well-being. Experimental deletion of Müller cells results in disorganization of retinal layers, photoreceptor degeneration, and malformation of the retinal vasculature (1). Moreover, recent studies on Müller cells in the pathologically altered retina clearly indicate that gene expression changes and functiol constraints in Müller cells, because of their response to tissue damage, are very likely to affect neuronal survival in the diseased retina (2–4). However, strikingly little is known about the mechanisms and modulatory factors of this Müller cell reaction termed Müller cell gliosis. Additionally, there is an ongoing discussion whether Müller cell gliosis has primarily detrimental or also beneficial effects on retinal neurons (5–7).

To answer these questions, there is an urgent need of in-depth, comprehensive characterization of Müller cell protein expression to better understand how they intimately interact with retinal neurons, microglia, and retinal vasculature. Modern techniques for determining expression profiles from biological samples have evolved into powerful, highly sensitive, quantitative tools that are extensively applied to generate huge sets of data. These techniques include proteomic methods such as mass spectrometry with ever-increasing sensitivity to analyze protein expression, translating gene expression into the effector level. Combined with a cell fractionation sample preparation approach, information about subcellular localization of proteins can be gained, enabling a better understanding of the underlying mechanisms.

Comprehensive proteomic data have been previously collected from whole retinal tissue samples (8–11), however, major limitations with respect to assigning altered protein expression levels to functional changes at cellular resolution remain. The retina comprises multiple highly specialized cell types, with neurons largely outnumbering Müller cells which make up only 1.5% of the cell population of the murine retina (12). To identify expression of Müller cell proteins, it is therefore inevitable and logical to reconsider current approaches and to switch from whole tissue expression analysis to (Müller) cell type-specific data generation.

To date, only very few studies have performed cell type-specific mRNA expression analysis of Müller cells. Enrichment of Müller cells from the adult retinal tissue is highly challenging because of their intricate and fragile morphology and huge cell size. Picking single Müller cells from dissociated murine retinal tissue under the microscope, Roesch *et al.* (13) performed single-cell microarrays analyses using very limited numbers of cells (2–5 cells per cell type). Another study reported microarray data from murine Müller glia that were enriched by fluorescence-activated cell sorting (FACS)^1^ (14). However, because FACS sorting severely distorts cell morphology by tearing apart cell processes leaving rounded Müller cells, this enrichment method is not well suited for proteomic studies because it likely alters detectable levels of proteins, especially proteins that allocate specifically to fragile cellular processes.

Protein expression data from Müller cells are so far restricted to studies of cultured cells (15, 16). Unfortunately, taking Müller glia into cell selective culture leads to their rapid dedifferentiation. A price for obtaining relatively pure Müller cells at high numbers, the cells gain stem cell-like characteristics after a few days in culture (15). Thus, expression profiles from cultured Müller glia do not adequately mirror those of Müller cells in native tissue. Accordingly, comprehensive data on protein expression from differentiated native Müller glia are missing completely to date.

We set out to first establish and validate a novel rapid method of isolating and enriching adult Müller cells from intact tissue, which yields sufficient cell numbers for proteomic profiling while keeping cells viable and fully intact morphologically as well as physiologically. From these preparations we collected a comprehensive data set of the proteome of native adult murine Müller cells. Because many Müller cell functions depend on cell-cell interactions occurring at the plasma membrane level, we additionally included a subcellular fractionation approach that enabled discrimination of proteins primarily residing in the cytosol or the nucleus from those allocated in the plasma membrane and thus generate for the first time a Müller cell surfaceome study. We consider these data a first important step to better understanding the complex crosstalk between various retinal cell types and Müller cells as key players in health and ultimately also under disease conditions.

## EXPERIMENTAL PROCEDURES

### 

#### 

##### Animals

All experiments were done in accordance with the European Communities Council Directive 86/609/EEC, and were approved by the local authorities. Animals were maintained with free access to water and food in an air-conditioned room on a 12-hour light-dark cycle. Adult (2–4 months old) C57Bl6/J mice were used for isolation of native Müller cells.

##### Müller cell isolation and MACS sorting

Isolated retinae were incubated with papain (0.2 mg/ml, Roche, Mannheim, Germany) in Ca^2+^-/Mg2+-free phosphate-buffered saline containing 11 mm glucose, pH 7.4, for 30 min at 37 °C, followed by several washing steps with saline. After short incubation in saline supplemented with DNase I (200 U/ml), the tissue was triturated in extracellular solution (ECS, that contained (mm) 135 NaCl, 3 KCl, 2 CaCl_2_, 1 MgCl_2_, 1 Na_2_HPO_4_, 10 HEPES, and 11 glucose, adjusted to pH 7.4 with Tris) to obtain isolated retinal cells. After centrifugation, the supernatant was removed and the cells were resuspended and incubated in ECS containing biotinylated hamster anti-CD29 (clone Ha2/5, BD Biosciences, Heidelberg, Germany) for 15 min at 4 °C. After washes in ECS and centrifugation, cells were taken up in ECS containing anti-biotin MicroBeads (Miltenyi Biotec, Bergisch Gladbach, Germany) and incubated for 10 min at 4 °C. After an additional washing step in ECS, cell populations were separated using MACS® cell separation large cell columns (Miltenyi Biotec) according to the manufacturer's recommendation. If microglia cells were isolated in addition to Müller cells, the retinal suspension was incubated with CD11b-microbeads (Miltenyi Biotec) for 15 min at 4 °C and positively selected using MACS® cell separation large cell columns (Miltenyi Biotec) before Müller cells were surface-labeled for MACS sorting.

10 μl from both samples (positive and negative fraction) were transferred to a counting chamber (Labor Optik, Friedrichsdorf, Germany) to assess information about obtained cell numbers using a standard protocol. Another 50 μl of each sample were fixed in 4% paraformaldehyde, washed in buffered saline, and then cell nuclei and Müller cells (using labeling against glutamine synthetase and CRALBP as marker protein) were fluorescently labeled on slide. We assessed the ratio of identified Müller cells (based on glutamine synthetase labeling and the intricate morphology of the cells) and total counts of cell nuclei to determine the purity of the respective cell subpopulations.

Remaining sample volumes were centrifuged and the cell pellet was prepared either for proteomic analysis or RNA extraction.

##### Subcellular Fractionation of Isolated Retinal Cell Types

The Müller cell enriched-samples contained 500,000 and 800,000 cells (representing two biological replicates) obtained by pooling isolated cell subpopulations from four retinae derived from two animals per replicate and <1 500,000 cells in the neuronal fraction, respectively. Cells were separated into cell surface fraction, nuclear fraction and a crude “cytosolic” fraction as follows. To achieve a specific biotinylation of sialylated surface proteins, a periodate oxidation and aniline-catalyzed oxime ligation-based cell surface labeling procedure (17) was performed on vital cells with cell membranes still being intact. The experiment was performed as described (18, 19). Briefly, after MACS sorting, cells were pelleted by centrifugation and resuspended in phosphate-buffered saline (PBS), pH 6.7, containing 11 mm glucose before the cells were transferred to the labeling solution (11 mm glucose, 500 μm aminooxy-biotin, 1 mm NaIO_4_, 0.94 mg/ml aniline, in PBS, pH 6.7) and incubated under constant rotation for 30 min at 4 °C. The reaction was stopped by adding glycerol to a final concentration of 1 mm and after additional washes in TBS (30 mm Tris, 150 mm NaCl) the cells were pelleted by centrifugation and resuspended in low-salt lysis buffer (10 mm Tris/HCl pH 7.6, 10 mm NaCl, 1% Nonidet P-40, 1× protease inhibitors) in order to keep the nuclei intact. After careful sonication, the nuclei were separated by centrifugation at 6000 × *g* for 10 min at 4 °C, briefly washed with 10 mm Tris/HCl pH 7.6/10 mm NaCl and stepwise resuspended in high-salt buffer (10 mm Tris/HCl pH 7.6, 500 mm NaCl) without or with 1% Triton X-100 and total protein content measured by a Bradford assay (Bio-Rad, Munich, Germany) following the manufacturer's instructions. Each 10 μg of extracted nuclear proteins were subjected to tryptic digest applying a modified FASP procedure (20) as follows. For protein reduction, 1 μl of 1 m DTT was added to the samples and incubated for 30 min at 60 °C. After cooling the samples to room temperature, the samples were diluted with UA buffer (8 m urea in 0.1 m Tris/HCl pH 8.5) and 10 μl of freshly prepared 300 mm iodacetamide solution were added for 30 min at room temperature in the dark. Samples were centrifuged through a 30 kDa cut-off filter device (PALL) and washed thrice with UA buffer and twice with 50 mm ammoniumbicarbonate (ABC). Proteins were digested in 40 μl of 50 mm ABC for 2 h at room temperature using 1 μg Lys-C (Wako Chemicals, Neuss, Germany) and for 16h at 37 °C using 2 μg trypsin (Sigma Aldrich, Taufkirchen, Germany). Peptides were collected by centrifugation and filters were washed with 20 μl 50 mm ABC/2% ACN. Samples were acidified with 0.5% trifluoroacetic acid (TFA) prior to mass spectrometric analysis.

The supernatant of the centrifuged cell lysate containing cytoplasmic and surface proteins was diluted fivefold with TBS and biotinylated proteins were extracted by binding to 80 μl (50% bead slurry) equilibrated strep-tactin superflow beads (suspension beads, IBA, Göttingen, Germany) at 4 °C for 2h. The flow-through containing the crude cytosolic fraction was measured for total protein content using a Bradford assay, and 10 μg were proteolysed applying a modified FASP procedure similar to the nuclear fraction (see above).

The beads with bound surface proteins were washed with TBS/0.2% Nonidet P-40 pH 7.4 and 0.5%SDS/TBS pH 7.4. Beads were incubated with 0.5%SDS/100 mm DTT/TBS pH 7.4 for 30 min at room temperature and washed with UC buffer (6 m urea/100 mm Tris-HCl, pH 8.5). Beads were incubated with UC buffer containing 50 mm iodoacetamide for 30 min at room temperature and then washed sequentially with UC buffer, 5 m NaCl, 100 mm Na_2_CO_3_ (pH 11.5), 50 mm Tris-HCl pH 8.5. Bound proteins were subjected to tryptic digest directly on the affinity matrix in 50 mm Tris-HCl pH 8.5 with 1 μg trypsin for 16h at 37 °C. Tryptic peptides were collected by centrifugation. Residual peptides were eluted with an additional elution in 50 mm Tris-HCl pH 8.5 and pooled with the first eluate. Beads were washed with 50 mm sodium phosphate pH 7.5 and glycopeptides were released using 500 Units PNGaseF (New England Biolabs) in 50 mm sodium phosphate pH 7.5 for 6 h at 37 °C. Glycopeptides were collected by centrifugation and residual peptides were eluted with 50 mm sodium phosphate pH 7.5. All eluates were pooled, acidified with TFA and analyzed on the OrbitrapXL.

##### iST Sample Preparation

There was an input of ∼200,000 cells from the Müller cell-enriched and 500,000 cells from the Müller cell-depleted fraction obtained by pooling cell subpopulations from two retinae derived from one animal per replicate; three biological replicates were analyzed. Cells were processed for LC-MS/MS with the in-StageTip (iST) method as described (21) with slight adaptations. Briefly, cells were lysed in lysis buffer (6 m GdmCl, 10 mm TCEP, 40 mm chloroacetamide, 100 mm Tris pH 8.5), boiled for 8 min at 95 °C and subsequently sonicated for 5 × 1 min using a waterbath sonicator (Elma Transsonic T310-H). Cell lysates were diluted 1:10 with dilution buffer (10% (v/v) ACN, 25 mm Tris pH 8.5) containing 1 μg Trypsin and Lys-C each and digested overnight at 37 °C. After digestion peptides were acidified to an end-concentration of 1% TFA. Prior to peptide loading to the StageTip, activation of material (14-gauge plug SDB-RPS, Empore^TM^, 3M Bioanalytical Technologies, Neuss, Germany) was performed by successively applying 50 μl acetone, isopropanol, methanol and 0.2% TFA (v/v) followed by centrifugation in between (1 min, 1000 × *g*). Acidified peptides were transferred to the StageTip and loaded onto activated SDB-RPS material by centrifugation for 1–2 min for up to 1500 × *g*. The loading procedure was repeated three times. Then the loaded StageTip was washed three times using 100 μl 0.2% TFA (v/v). Peptide elution was performed in four fractions by successive application of 60 μl elution buffers 1–4 (supplemental Table S7) and centrifugation for 1–2 min for up to 1500 × *g*. Eluates were collected and evaporated using a SpeedVac centrifuge. Peptides were resuspended in 50 μl loading buffer (2% ACN, 0.5% TFA), briefly sonicated and used for LC-MS/MS analysis.

##### Mass Spectrometry

LC-MS/MS analysis was performed as described previously (22, 23) on an LTQ OrbitrapXL (Thermo Fisher Scientific Inc., Waltham, MA). Approximately 0.5 μg per sample were automatically loaded to the HPLC system. A nano trap column was used (300 μm inner diameter × 5 mm, packed with Acclaim PepMap100 C18. 5 μm, 100 Å; LC Packings, Sunnyvale, CA) before separation by reversed phase chromatography (PepMap, 25 cm, 75 μm ID, 2 μm/100 Å pore size, LC Packings) operated on a RSLC (Ultimate 3000, Dionex, Sunnyvale, CA). Peptides were eluted with the following gradient of increasing ACN concentrations in 0.1% formic acid over 170 min: 135 min of 6% to 31% followed by 10 min from 31% to 72% ACN. Between each gradient the ACN in 0.1% FA concentration was set back to starting conditions for 20 min. From the high resolution MS prescan, the 10 most abundant peptide ions were selected for fragmentation in the linear ion trap if they were at least doubly charged and if they exceeded an intensity of at least 200 counts, with a dynamic exclusion of 60 s. During fragment analysis, a high-resolution (60,000 full width at half-maximum) MS spectrum was acquired with a mass range from 300 to 1500 Da.

##### Label-free Analyses (Including Database Search and Protein Identification)

The acquired spectra of the different samples were loaded and analyzed using Progenesis QI software for proteomics (Version 2.0, Nonlinear Dynamics, Waters, Newcastle upon Tyne, U.K.) for label-free quantification as previously described (23). The profile data of the MS scans were transformed into peak lists with respective *m*/*z* values, intensities, abundances, and *m*/*z* width. MS/MS spectra were transformed similarly and then stored in peak lists comprising *m*/*z* and abundance. Using one sample as reference, the retention times of the other samples were aligned by automatic alignment to a maximal overlay of the 2D features. Features with one or more than seven charges were masked at this point and excluded from further analyses. After alignment and feature exclusion, samples were allocated to their respective experimental groups (Müller cell-enriched *versus* -depleted fractions), and raw abundances of all features were normalized. Normalization corrects for factors resulting from experimental variation and was automatically calculated over all features in all samples to correct for technical variation.

All MS/MS spectra were exported from the Progenesis QI software as Mascot generic files (mgf) and used for peptide identification with Mascot (version 2.5) using the Ensembl Mouse protein database (mus musculus; release 75, containing 51772 sequences). Search parameters used were 10 ppm peptide mass tolerance, 0.6 Da fragment mass tolerance, one missed cleavage allowed, carbamidomethylation was set as fixed modification, and methionine oxidation and deamidation of asparagine and glutamine as variable modifications. Mascot integrated decoy database search was set to a false discovery rate (FDR) of 1% when searching was performed on the concatenated mgf files with a percolator ion score cut-off of 13 and an appropriate significance threshold p. Identifications were re-imported into Progenesis QI. For quantification, all unique peptides of an identified protein were included and the total cumulative normalized abundance was calculated by summing up the abundances of all unique peptides allocated to the respective protein. No minimal thresholds were set neither for the method of peak picking nor selection of data to use for quantification. Statistical analysis was performed using transformed (“log-like” arcsinh(.)function) normalized abundances for one-way analysis of variance (ANOVA) calculations of all detected features. ANOVA values of *p* < 0.05 and additionally regulation of ≥twofold or ≤ 0.5-fold were regarded as significant for all further results.

Two technical replicates were measured for every type of sample preparation to verify robust experimental reproducibility with a mean coefficient of variation (CV) of 8.44% for the iST and 9.58% for the subcellular fractionation approach (see supplemental Fig. S2). Data from two (subcellular fractionation) or three (iST approach) biological replicates are presented in the results part (Figs. 2 and 3, supplemental Fig. S3).

The mass spectrometry proteomics data have been deposited to the ProteomeXchange Consortium (24) via the PRIDE partner repository with the data set identifier PXD002247 and 10.6019/PXD002247.

##### Heat Maps

Heat maps of significantly altered proteins were generated for the two different sample preparation methods separately, using the heat map function in the Excel (Microsoft) add-in XLStat including the OMICS module (Addinsoft). Normalized protein abundances were used for independent clustering of samples and proteins, applying hierarchical clustering based on Euclidian distances. The corresponding colored matrices are displayed in Fig. 3, with high abundant proteins in the respective sample displayed in green and low abundant proteins in red.

##### Classification of Identified Proteins

In order to assess subcellular localization of identified proteins, FASTA files were generated and submitted to Phobius, a combined transmembrane topology and signal peptide predictor (http://phobius.sbc.su.se/index.html, (25)). Proteins were accordingly classified into three categories depending on the presence of an exclusive secretory signal peptide (cell surface proteins), a secretory signal peptide in combination with at least one transmembrane domain (plasma membrane proteins) or no signal peptide with or without (a) transmembrane domain(s) (other proteins).

##### Pathway Enrichment Analyses and Network Generation

Pathway enrichment analysis was performed with Genomatix Generanker (http://www.genomatix.de, Genomatix). Gene names of significantly different proteins were uploaded and corresponding overrepresented pathways and processes are given in Tables I and II and supplemental Tables S3, S4, S5 and S6.

**Table I:** Pathway enrichment analyses performed with data sets from iST sample preparation. Top hits of overrepresented pathways and functions for proteins significantly higher expressed in the Müller cell-enriched fraction compared to the Müller-cell depleted neuronal fraction are listed with their respective enrichment score (*p value*). The full results lists are found in supplemental Tables V (enriched Müller cells) and 3 (Müller-cell depleted retinal neurons)

Enriched Müller cells–iST data	p value	Müller cell-depleted retinal neurons - iST data	*p* value
*Signal transduction*		*Signal transduction*	
Paxillin	3.97E-08	Visual signal transduction: Rods	2.64E-12
Endocytic	9.54E-08	Visual signal transduction: Cones	9.01E-07
Trafficking	1.60E-07	Mechanisms of transcriptional repression by DNA methylation	2.24E-05
Small GTP binding protein rac	4.06E-07	Mechanism of protein import into the nucleus	2.90E-03
Agrin in postsynaptic differentiation	2.74E-07	Guanylate cyclase	3.79E-05
CDC42 signaling events	5.05E-06	Nuclear receptor subfamily 2. group e. member 3	2.70E-04
Integrin signaling pathway	1.23E-05	DNA repair	2.84E-04
RAC1 signaling pathway	6.59E-05	G-protein coupled receptor kinase	5.99E-04
		Cellular growth	1.34E-03
		Telomerase reverse transcriptase	9.14E-03
*Molecular function (GO)*		*Molecular function (GO)*	
Cytoskeletal protein binding	3.98E-25	Poly(A) RNA binding	1.61E-40
Anion binding	7.39E-08	RNA binding	8.88E-38
Phospholipase inhibitor activity	1.18E-07	Heterocyclic compound binding	9.20E-19
Lipid binding	1.89E-07	Nucleotide binding	3.75E-09
Calmodulin binding	4.98E-07	NADH dehydrogenase activity	1.91E-08
Calcium ion binding	7.81E-07	Oxidoreductase activity	4.35E-07
Actin filament binding	8.92E-07	Unfolded protein binding	8.58E-07
Phospholipid binding	9.23E-07	Nucleoside-triphosphatase activity	1.49E-06
Lipase inhibitor activity	1.17E-06	Ribonucleoprotein complex binding	4.11E-06
Ion channel binding	4.81E-06	Structure-specific DNA binding	5.55E-06
Cell adhesion molecule binding	8.11E-06	ATPase activity, coupled	6.85E-06
		Photoreceptor activity	6.05E-04
		siRNA binding	8.07E-03
*Cellular components*		*Cellular components*	
Membrane-bounded vesicle	5.54E-55	Intracellular organelle part	2.01E-39
Extracellular vesicular Exosome	4.04E-53	Nuclear part	1.84E-30
Adherens junction	1.61E-33	Ribonucleoprotein complex	2.42E-30
Cytoplasm	1.20E-31	Membrane-bounded organelle	3.93E-27
Cytoskeleton	7.23E-19	Nucleoplasm	1.93E-26
Cell projection	1.70E-17	Spliceosomal complex	1.66E-25
Actin cytoskeleton	4.55E-16	Mitochondrial part	2.79E-24
Cytoplasmic membrane-bounded vesicle	1.43E-13	Nucleolus	4.21E-20
Plasma membrane	2.32E-13	Mitochondrial inner membrane	1.47E-15
Cytoplasmic vesicle	3.02E-13	Photoreceptor outer segment	3.16E-12
		Ciliary membrane	6.93E-12
*Biological processes*		*Biological processes*	
Actin filament-based process	1.49E-21	RNA splicing	1.90E-31
Actin cytoskeleton organization	2.96E-18	mRNA processing	5.54E-31
Transport	6.16E-13	mRNA splicing. via spliceosome	1.15E-28
Regulation of biological quality	1.13E-12	Cellular respiration	7.87E-18
Establishment of localization	1.60E-12	Nucleobase-containing compound Metabolic process	2.59E-17
Muscle contraction	1.75E-12	Cellular nitrogen compound Metabolic process	1.48E-16
Response to wounding	3.71E-11	Ribonucleoprotein complex biogenesis	3.09E-15
Hemostasis	8.01E-10	Detection of light stimulus	1.77E-14
Regulation of neurotransmitter levels	6.95E-07	Energy derivation by oxidation of organic compounds	2.74E-13
Phagocytosis	7.46E-07	RNA splicing	1.90E-31
*Tissues*		*Tissues*	
Neuroglia	2.53E-14	Hela cells	5.47E-20
Tissue membrane	3.78E-14	Photoreceptors	2.05E-17
Structure of cortex of kidney	1.04E-11	Rod photoreceptors	4.23E-17
Muscle cells	9.26E-10	Retinal cone	6.11E-11
Müller cells	1.51E-09	Retina	7.01E-10

**Table II:** Pathway enrichment analyses performed with data sets from subcellular fractionation sample preparation. Top hits of overrepresented pathways and functions for proteins significantly higher expressed in the Müller cell-enriched fraction compared to the Müller-cell depleted neuronal fraction are listed with their respective enrichment score (*p value*). The full results lists are found in supplemental Tables S6 (enriched Müller cells) and S4 (Müller-cell depleted retinal neurons)

Enriched Müller cells–Subcellular fractionation data	p value	Müller cell-depleted retinal neurons–Subcellular fractionation data	*p* value
*Signal transduction*		*Signal transduction*	
Integrin	1.06E-06	Visual signal transduction: rods	8.87E-11
Extrinsic prothrombin activation pathway	1.20E-04	Visual signal transduction	1.81E-07
Paxillin	5.28E-04	Visual signal transduction: cones	4.36E-06
Nitric oxide	5.98E-04	Endocytotic role of ndk phosphins and dynamin	8.44E-04
T-cell receptor cd3 complex	7.22E-04	Casein kinase 2	8.91E-04
Protein kinase c	9.70E-04	Guanylate cyclase	1.25E-03
p21(cdkn1a) activated kinase	1.11E-03	Second messenger cGMP	1.56E-03
Erk and PI-3 kinase are necessary for collagen binding in corneal epithelia	1.50E-03	G-protein coupled receptor kinase	1.98E-03
Tissue inhibitor of metalloproteinase	1.86E-03	Dual specificity tyrosine phosphorylation regulated kinase	2.51E-03
Focal adhesion kinase 1	2.46E-03		
Serum/glucocorticoid regulated kinase	2.55E-03		
Tyrosine protein kinase src	5.07E-03		
Angiopoietin receptor tie2-mediated signaling	5.45E-03		
Calcium	8.82E-03		
*Molecular function (GO)*		*Molecular function (GO)*	
Substrate-specific transmembrane transporter activity	1.11E-07	Monovalent inorganic cation transmembrane transporter activity	2.14E-08
Ion transmembrane transporter activity	1.85E-07	Hydrogen ion transmembrane transporter activity	1.14E-07
Cation transmembrane transporter activity	7.85E-07	NADH dehydrogenase activity	8.86E-07
Carboxylic acid transmembrane transporter activity	1.25E-06	Catalytic activity	3.40E-06
Cell adhesion molecule binding	3.10E-06	Oxidoreductase activity. acting on NAD(P)H, quinone or similar compound as acceptor	5.66E-06
Sodium ion transmembrane Transporter activity	6.41E-05	ATPase activity. coupled to transmembrane movement of substances	2.63E-05
Anion:cation symporter activity	1.37E-04	Nucleoside-triphosphatase activity	3.63E-05
Solute:sodium symporter activity	1.49E-04	Pyrophosphatase activity	7.72E-05
Phospholipase A2 inhibitor activity	1.78E-04	Poly(A) RNA binding	1.04E-04
Gamma-aminobutyric acid:sodium symporter activity	1.78E-04	Electron carrier activity	2.00E-04
Actin binding	2.40E-04	Photoreceptor activity	3.77E-04
ATPase activity, coupled to transmembrane movement of ions. phosphorylative mechanism	5.83E-04		
Glutamate binding	1.30E-03		
*Cellular components*		*Cellular components*	
Plasma membrane	1.65E-15	Photoreceptor outer segment	1.84E-12
Extracellular vesicular exosome	2.75E-15	Organelle membrane	2.91E-12
Cell periphery	4.86E-15	Neuron part	1.94E-11
Membrane-bounded vesicle	9.48E-14	Ciliary membrane	8.84E-11
Extracellular region	4.05E-10	Photoreceptor outer segment membrane	1.43E-09
Sarcolemma	2.05E-09	Synaptic vesicle membrane	1.45E-08
Adherens junction	5.34E-09	Photoreceptor disc membrane	1.06E-07
Focal adhesion	5.61E-09	Respiratory chain	1.47E-07
Membrane raft	2.02E-07	Cell projection	4.45E-07
T-tubule	1.14E-06	Mitochondrial membrane	4.88E-07
Filopodium	2.15E-06		
Sodium:potassium-exchanging ATPase complex	2.01E-05		
Secretory granule	2.03E-05		
Cytoplasm	7.31E-05		
*Biological processes*		*Biological processes*	
Localization	4.45E-10	Phototransduction	2.16E-15
Cell-substrate adhesion	2.18E-09	Generation of precursor metabolites and energy	2.26E-13
Ion transport	6.99E-09	Rhodopsin mediated signaling pathway	4.74E-13
Transport	1.85E-08	Energy derivation by oxidation of organic compounds	1.59E-12
Establishment of localization	3.79E-08	Respiratory electron transport chain	5.96E-12
Cell-matrix adhesion	4.19E-08	Retina development in camera-type eye	1.75E-08
Carboxylic acid transport	1.38E-07	Mitochondrial electron transport, NADH to ubiquinone	7.64E-08
Hemostasis	1.80E-07	Neurotransmitter transport	1.48E-07
Wound healing	5.81E-07	Hydrogen ion transmembrane transport	1.84E-07
Glutamine family amino acid Metabolic process	2.58E-05	Purine ribonucleoside monophosphate metabolic process	1.90E-07
Cation transport	3.40E-05	Regulation of G-protein coupled receptor protein signaling pathway	4.88E-06
Anion transmembrane transport	3.96E-05	Photoreceptor cell differentiation	5.46E-06
Homotypic cell–cell adhesion	4.81E-05	Neurotransmitter secretion	6.86E-06
Cation transport	4.45E-10	mRNA splicing via spliceosome	2.89E-05
Anion transmembrane transport	2.18E-09		
Homotypic cell–cell adhesion	6.99E-09		
*Tissues*		*Tissues*	
Astrocytes	3.95E-12	Rod photoreceptors	1.50E-18
Neuroglia	3.65E-09	Retinal cone	3.46E-17
Epithelial cells	6.44E-08	Photoreceptors	2.57E-16
Müller cells	3.02E-06	Retina	6.75E-16
Outer limiting membrane	3.84E-06	Pinealocyte	3.57E-14

For protein network generation the Genomatix Pathway System (GePS, http://www.genomatix.de, Genomatix) was used. Corresponding gene names of significantly different proteins were uploaded and networks were created using Mus musculus as the organism with literature mining conducted at the sentence level.

##### qRT-PCR

The RNeasy Micro Kit (Qiagen, Hilden, Germany) was applied according to the manufacturer's instructions to isolate total RNA from retinal tissue or respective isolated cell subpopulations. An on-column DNase digestion step was included to reliably remove genomic DNA contamination (Roche). First-strand cDNAs from 50 ng of total RNA were synthesized using the RevertAid H Minus First-Strand cDNA Synthesis Kit (Fermentas by Thermo Fisher Scientific, Schwerte, Germany). Candidate gene expression levels were validated performing qRT-PCR using cDNA derived from whole retinal extracts, Müller cell-enriched and Müller cell-depleted fractions with the TaqMan hPSC Scorecard™ Panel (384 well, ViiA7, Life Technologies, Darmstadt, Germany) according to the company's guidelines and using its cloud-based online analysis software.

##### Immunofluorescent Labeling of Retinal Samples

A drop of each fixed retinal cell suspensions obtained from the cell isolation procedure was allowed to dry on a glass slide, was then blocked in 5% normal goat serum plus 0.3% Triton X-100 plus 1.0% DMSO in saline for 30 min at room temperature. After several washing steps in buffered saline, cells were incubated with the primary antibodies (mouse anti-glutamine synthetase, 1:1000, Merck Millipore, Darmstadt, Germany; rabbit anti-protein kinase alpha (PKCα), 1:300, Santa Cruz, Heidelberg, Germany; rabbit anti-Prdx6, 1:100, Abcam, Cambridge, UK; mouse anti-rhodopsin, 1:500, kindly provided by Dr. Robert Molday, University of British Columbia, Vancouver; rabbit anti-CRALBP, 1:200, Protein Technologies, Tucson, AZ) for 1 h at room temperature, washed in in 1% bovine serum albumin and were subsequently incubated with the secondary antibodies (Cy2-conjugated goat anti-mouse, 1:200; Cy3-conjugated goat anti-rabbit, 1:200; both from Dianova, Hamburg, Germany) for 1 h at room temperature. Finally, cells were washed again in buffered saline and embedded in ProLong® Antifade medium containing DAPI (Life Technologies) for nuclear labeling. For relative quantification of protein expression levels based on fluorescence signal intensities of CRALBP, Prdx6, and rhodopsin labelings, ImageJ (U.S. National Institutes of Health, Bethesda, MD) analysis was performed measuring the mean fluorescence intensity across each complete cell normalized to the area of the cell. Values were background corrected.

After enucleation, eyes were fixed in 4% paraformaldehyde for 24 h, washed in buffered saline and afterward incubated in 30% sucrose in buffered saline overnight for cryoprotection. 10-μm thick slices were cut by using a microtome cryostat (Microm, Walldorf, Germany) from the tissues that were embedded in Neg50 (Thermo Fisher Scientific, Schwerte, Germany). The slices were incubated in 5% normal goat serum plus 0.3% Triton X-100 plus 1.0% DMSO in saline for 2 h at room temperature and, subsequently, in the primary antibodies (rabbit anti-Prdx6, 1:100, Abcam; or rabbit anti-Folh1, 1:100, Cell Signaling, Cambridge; mouse anti-glutamine synthetase, 1:500, Merck Millipore) overnight at 4 °C. After washing in 1% bovine serum albumin, the secondary antibody (Cy2-conjugated goat anti-rabbit, 1:200, Dianova) was applied overnight at 4 °C in 1% bovine serum albumin. Cell nuclei were labeled with DAPI (1:1000; Life Technologies). As control, slices were stained without the primary antibody; no unspecific labeling was observed following incubation with the secondary antibody alone (not shown). Images were taken with custom-made VisiScope CSU-X1 Confocal System (Visitron Systems, Puchheim, Germany) equipped with high-resolution sCMOS camera (PCO AG, Kehlheim, Germany).

##### Western Blot

After separation of cell populations via MACS, cells were lysed in standard RIPA buffer (including 1 mm phenylmethylsulfonyl fluoride and supplemented with 1× protease inhibitor mixture, Roche) and protein concentrations were determined with the Bio-Rad DC Protein Assay (Bio-Rad, Munich, Germany) according to the manufacturer's instructions. Equal amounts of total protein per sample were separated by SDS-polyacrylamide gel electrophoresis and transferred onto polyvinylidenfluoride membranes (PVDF; Merck Millipore, Darmstadt, Germany) followed by incubation in 5% nonfat dry milk in PBS, containing 0.2% Tween 20, for 1 h at room temperature. Target detection was performed by overnight incubation at 4 °C with primary antibodies: rabbit anti-Prdx6, 1:1000 (Abcam, Cambridge, UK), mouse anti-actin (1:10.000, Sigma-Aldrich, Munich, Germany) followed by incubation with secondary antibodies (peroxidase-conjugated anti-rabbit or anti-mouse IgG; 1:10.000; Calbiochem/Merck, Darmstadt, Germany) for 1 h at room temperature. Protein labeling was visualized by enhanced chemiluminescence using the Odyssey® Fc Imager (LI-COR Biotechnology, Bad Homburg, Germany). For visualization of total transferred proteins on the PVDF membranes a common Ponceau S staining protocol was performed before the blocking step.

##### Imaging of Reactive Oxygen Species (ROS) Formation in Various Retinal Cell Types

Acutely isolated retinae were papain digested and mechanical triturated to obtain a single cell retinal cell solution as described above. Retinal cells were transferred to a recording chamber mounted on the stage of an upright confocal laser scanning microscope (LSM 510 Meta; Zeiss, Oberkochen, Germany). The cells were loaded with the ROS-sensitive fluorescent indicator CM-H2DCFDA diacetate (30 μm; Molecular Probes, Invitrogen, Eugene, OR) for 30 min at 37 °C. Stem solution of CM-H2DCFDA was prepared in DMSO. Thereafter, the cells were continuously superfused with extracellular solution at a flow rate of 2 ml/min, and recordings were made with an Achroplan 63x/0.9 water immersion objective. The pinhole was set at 172 μm; the thickness of the optical section was adjusted to 1 μm. CM-H2DCFDA was excited at 488 nm with an argon ion laser, and emission was recorded through a 505–530 nm bandpass filter. The recording chamber was continuously perfused by using a gravity-fed system with multiple reservoirs; H_2_O_2_ (100 μm or 40 mm) was applied by rapid change of the perfusate. The bathing solution in the recording chamber was totally changed within ∼15 s. The extracellular solution consisted of (in mm) 136 NaCl, 3 KCl, 2 CaCl_2_, 1 MgCl_2_, 10 HEPES, and 11 glucose, adjusted to pH 7.4 with Tris. To avoid photoactivation of CM-H2DCFDA pictures were taken at intervals of 30 s. The slope of a minor constant increase in CM-H2DCFDA fluorescence intensity because of photoactivation by the laser pulse was determined in baseline recordings for 3 min to allow later normalization to the latter. Thereafter, 100 μm of freshly dissolved H_2_O_2_ in ECL was applied for 15 min. Images were analyzed with the LSM 510 software. Alterations of CM-H2DCFDA fluorescence are presented as F/F0. The different cell types (bipolar cells, Müller cells, photoreceptor cells) where identified because of the distinct morphology.

##### Statistics

Data are expressed as means ± S.E. (S.E.). Statistical analysis was made using Prism (Graphpad Software, San Diego, CA); significance was determined by the nonparametric Mann-Whitney *U* test.

## RESULTS

### 

#### 

##### Characterization of MACS-based Müller Cell Isolation Form Murine Retinae

**Fig. 1.**
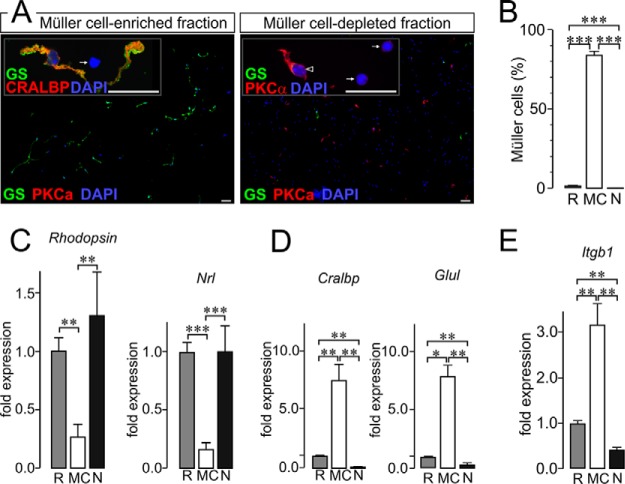
**Itgb1-MACS allows efficient enrichment of morphologically intact Müller glia cells from murine retinae.** *A*, Glutamine synthetase (GS) and cellular retinaldehyde binding protein (CRALBP) served as markers for Müller cells, PKCα labeling delineates rod photoreceptor bipolar cells (arrowhead in the inset marks a bipolar cell at higher magnification) and DAPI stains all nuclei in the sample. Note the excellent preservation of Müller cell morphology also depicted in the insert showing an enlarged view of an isolated Müller cell. Photoreceptor nuclei can easily discerned from that of all other cell types owing to their unique chromatin structure with tightly packed central chromatin core (arrows). Scale bars, 25 μm. *B*, The percentage of Müller cells in each cellular subpopulation was determined on the basis of the immunolabelings. The number of cells positive for the Müller cell marker glutamine synthetase was put into relation to the total cell number (number of DAPI-positive cell nuclei). Each bar represents values from 8 independent experiments for each of which retinae from two animals were pooled. ***p < 0.001. *C–E*, Quantitative RT-PCR experiments on cDNA transcribed from total RNA isolated from whole retinae (R), Müller cell enriched (MC) and Müller cell-depleted neuronal (N) cell subpopulations. *C*, Photoreceptor-specific transcripts rhodopsin and Nrl were present to a significantly lesser extent in the Müller cell-enriched samples than in whole retinal or Müller cell-depleted neuronal samples. *D*, Müller cell-specific transcripts (Cralbp and glutamine synthetase (Glul)) were strongly enriched in the Müller cell fraction and almost completely absent in the Müller cell-depleted fraction. *E*, Integrin β1 (Itgb1) is expressed at significantly higher levels in Müller glia than in retinal neurons. *B–D,* Values are given as ±S.E. and include results from five independent qRT-PCR experiments (*n* = 5). For sample generation cells from four retinae derived from two animals were pooled. ***p < 0.001, **p < 0.01, *p < 0.05.

Investigating the role of (Müller) glial cells in adult nervous tissue under various conditions including disease models requires the establishment of a method that allows efficient enrichment of glial cells from adult (retinal) tissue. We established a protocol based on magnetic-activated cell sorting (MACS) and the use of an antibody directed against Itgb1 as a surface marker of Müller cells. Immunolabeling of Müller and bipolar marker genes (glutamine synthetase, cellular retinaldehyde binding protein (CRALBP) and PKCα, respectively) of the MACS sorted cell populations gave a first impression of an efficient enrichment of Müller cells (Fig. 1*A*). Of note, we observed an excellent preservation of the long Müller cell stem processes and their highly specialized membrane protrusions in ∼80% of the cells. This is of highest relevance as the aim of a comprehensive analysis of their proteome, and more specifically their surfaceome, is of course beneficial for all other applications. Enrichment rates from ∼1.5% Müller cells in the retinal cell suspensions to up to 90% after MACS (60-fold) could be achieved and were confirmed by quantification of respective cell types in the immunolabelings (Fig. 1*B*). Of note, Müller cells were almost completely depleted from the original retinal cell suspension leaving a 99.7% pure neuronal fraction. Taken from immunolabelings of the cell fractions, the residual cell population contaminating the enriched Müller cell fraction (∼10%) are mainly photoreceptors based on quantification by their specific chromatin structure visualized by the DAPI labeling (52.5 ± 14.1% of contaminating cells are photoreceptors; *n* = 7 experiments). Neither bipolar cells nor ionized calcium binding adapter molecule 1 (Iba1)-positive microglia cells could be found in the Müller cell fraction (data not shown).

Validation of the enrichment using quantitative RT-PCR showed differential expression patterns between known Müller cell- and photoreceptor-specific genes, as expected in properly separated cellular subpopulations (Fig. 1*C*, 1*D*). These experiments also confirmed a minor contamination of the Müller cell fraction with photoreceptors (Fig. 1*C*). The expression of Müller cell markers in the neuronal fraction was almost completely absent (Fig. 1*D*) which is in line with the results from the cell quantification based on immunofluorescent labelings (Fig. 1*B*). Additionally, we tested whether enhanced expression of the surface marker Itgb1 could be detected in the Müller cell-enriched cell population; the expected expression levels were found in the respective cell populations (Fig. 1*E)*.

To ensure that our MACS based approach yielded sufficient cell counts for in-depth proteomic profiling, we evaluated the efficiency of the method for isolation of the total Müller cell population from an adult murine retina. Because Müller cells are not suited for automated cell counters because of their huge cell size (up to 200 μm *in vivo* and after isolation still reaching a length of up to 100 μm) we set up estimates of obtained cell numbers using a counting chamber. Previous studies suggest that the murine retina roughly comprises 230,000 Müller cells (12). We isolated on average 150,000 Müller cells from one murine retina, which equals 65% efficiency.

In sum, these data confirmed the suitability of this protocol in terms of an excellent purification of the various isolated cell types and the yield of cell counts to go for the planned comparative proteomic approach.

##### Comprehensive Proteomic Profiling by in-StageTip Digestion and Cellular Subfractionation Based on Surface Biotinylation for Sample Preparation

We applied two recent methods of sample preparation for high resolution mass spectrometry coupled to liquid chromatography (LC-MS/MS) to identify differentially expressed proteins in Müller cells and retinal neurons. Both methods are well optimized for in depth proteomic coverage with minimal sample input and thus well suited for analyzing limited amounts of acutely isolated Müller cells from mouse retina. First, we applied the iST method recently published by Kulak *et al.* (21) that eliminates sample contamination and limits sample loss throughout the preparation process, thereby enabling most sensitive proteomic profiling. Second, we combined *in situ* biotinylation of surface proteins (17) and subcellular fractionation as described earlier (18, 19) with mass spectrometry—a method that apart from identifying and quantifying proteins also yields valuable spatial information on the subcellular localization of the respective protein. A necessary prerequisite for successful performance of this method was efficient biotinylation of proteins residing exclusively on the cell surfaces of isolated cell populations. In control experiments, using detection of biotinylation with FITC-coupled streptavidin, the enriched cell populations demonstrated a robust delineation of cell membranes which was absent if the cells were not biotinylated (supplemental Fig. S1).

**Fig. 2.**
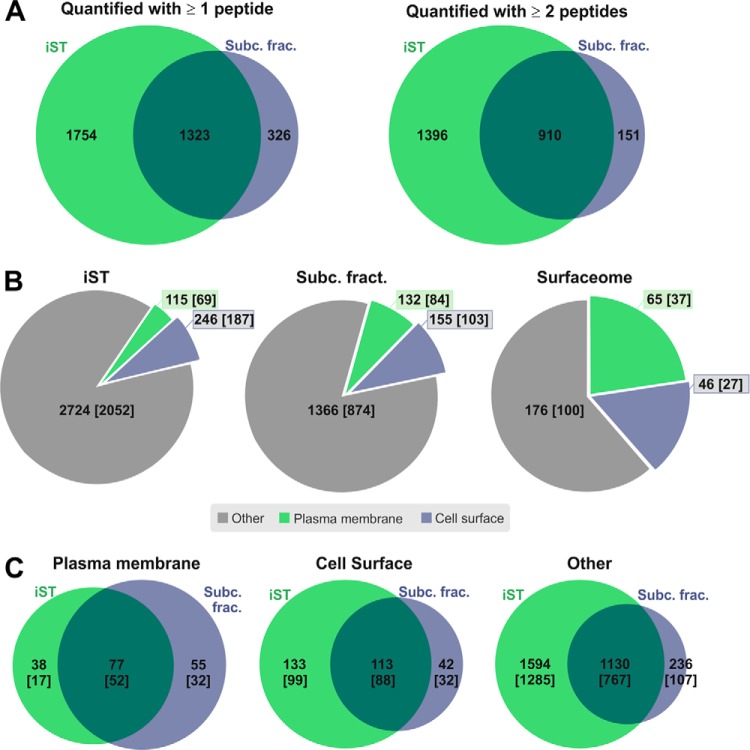
**Total protein identifications from samples prepared by iST and subcellular fractionations.** A, Numbers of protein identifications from iST (light green) *versus* subcellular fractionation method (blue) including one peptide hits (left venn diagram) and excluding one peptide hits (right venn diagram). Numbers of overlapping IDs are also indicated (dark green). B, All identified proteins from both fractionation approaches (values in brackets indicate proteins quantified with two or more peptides) are analyzed for subcellular localization by prediction of signal peptides for secretion and number of transmembrane domains. Protein IDs are categorized into: “Plasma Membrane” if a secretion signal peptide and at least one transmembrane domain were predicted (green), “Cell surface” if only a secretion peptide, but no transmembrane domain was predicted (blue). All other proteins are included under “Other” (gray). C, Identified protein numbers approaches (values in brackets indicate proteins quantified with two or more peptides) allocated to the three localization categories “Plasma Membrane,”, “Cell surface,” and “Other” are compared between the iST fractionation (green) and the subcellular fractionation (blue).

Applying the iST approach to Müller cell-enriched and -depleted fractions yielded a total of 3077 protein identifications with 2306 proteins identified and quantified with two or more peptides (Fig. 2*A* and supplemental Tables. S1, S8), with a mean technical CV of 8.44% (supplemental Fig. S2). In contrast, the subcellular fractionated samples (Müller cell-enriched and -depleted fractions) yielded lower protein numbers (1649 total proteins with 1061 proteins identified and quantified with two or more peptides) (Fig. 2*A* and supplemental Tables. S2, S8), with a comparable mean technical CV of 9.58% (supplemental Fig. S2). Although the majority of identified proteins overlapped between the two experimental approaches, significant numbers of proteins were exclusively identified with the iST approach (1754 proteins with 1396 proteins identified with two or more peptides) and fewer with the subcellular fractionation approach (326 proteins with 151 proteins identified with two or more peptides) (Fig. 2*A*). Because the subcellular fractionation approach included specific enrichment of cell surface and plasma membrane molecules, we analyzed and compared the yield of those proteins, which are often underrepresented in proteomic data sets. The subcellular fractionation approach yielded a total of 287 potential cell surface and plasma membrane proteins. 132 of these identifications which represent 17.3% of all identified proteins, contained a signal peptide for secretion and at least one transmembrane domain, and are thus designated plasma membrane proteins; 155 identifications contained only a predicted signal peptide for secretion and no transmembrane domain, and are designated cell surface proteins (Fig. 2*B*). The proportion of cell surface and plasma membrane proteins within the biotinylated sub-fraction excluding the nuclei and unlabeled intracellular proteins was 38.7% (with 65 plasma membrane proteins and 46 cell surface proteins, Fig. 2*B*). Within the proteins identified with the iST approach, 361 identifications are predicted to reside on the cell surface (with 115 plasma membrane protein identifications and 246 cell surface protein identifications), which represent 11.7% of total identifications in this data set.

In a next step, we investigated whether subcellular fractionation combined with surface biotinylation was superior in detecting proteins associated with the cell surface or membranes *per se*. Accordingly, we assessed to what extent presumable plasma membrane proteins (containing a signal peptide for secretion and at least one transmembrane domain predicted by *in silico* Phobius analysis (26)), cell surface proteins (with predicted signal peptide for secretion an no transmembrane domain) and those proteins fulfilling none of these criteria were identified with the two different approaches. Best congruence was observed for proteins with no predicted localization at the cell surface for which only 17.3% (236 proteins) identified by subcellular fractionation were not found in the iST analysis (Fig. 2*C*). The percentages of proteins exclusively found in the surface fractionation for plasma membrane proteins (41.7%, representing 55 protein identifications) and for cell surface proteins (27.1%, 42 proteins) were considerably higher indicating that our subcellular fractionation approach was more sensitive to detect membrane-associated proteins compared with the iST protocol (Fig. 2*C*).

**Fig. 3.**
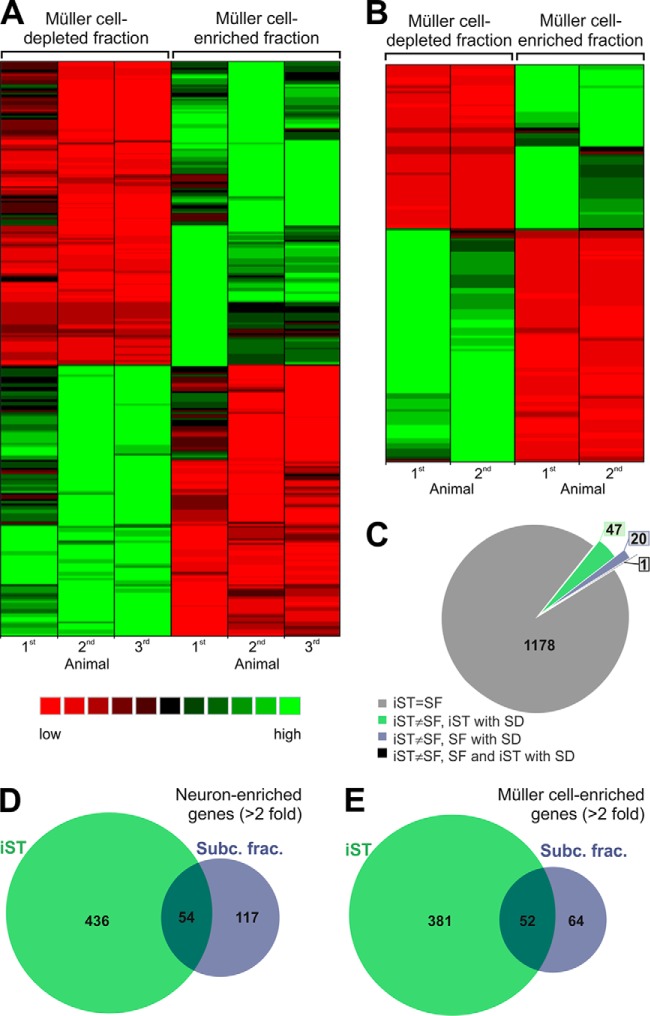
**Comparison of protein identifications from Müller cell-enriched and -depleted samples.** *A, B*, Heatmaps of hierarchical cluster analysis of significantly differently expressed proteins in Müller cell-enriched and -depleted fractions. Proteins and samples were clustered based on the respective protein abundances applying hierarchical clustering based on Euclidian distances. The corresponding heatmap is shown, with highly abundant proteins presented in green and low abundant proteins in red in the respective samples of (*A*) the iST approach or (*B*) the subcellular fractionation. *C*, Here we concentrated on proteins identified by both approaches (iST, subcellular fractionation (SF)) and determined whether the enrichment profiles of each protein found in the two approaches were in agreement with each other. For the vast majority of proteins, both approaches yielded a similar enrichment pattern (*gray*). Additionally, there were few proteins for which only one of the methods determined a significant difference (S.D.) in the expression level comparing both cell populations and the other method implicated an inverse enrichment, but did not reach significance (green, purple). Only one protein (black) was found to be significantly in enriched in Müller cells with one approach and the other approach indicated a significant enrichment in the neuronal fraction. *D*, *E*, Venn diagrams illustrating the overlapping and unique proteins identified with iST and subcellular fractionation of the Müller cell-depleted (neuronal) fraction (*D*) and the Müller cell enriched fraction (*E*). Threshold for enrichment to a specific fraction were set to ≥twofold enrichment and *p* < 0.05 (ANOVA of normalized cumulative peptide abundances).

In order to extract Müller cell expressed proteins from both data sets, we applied label-free quantifications and compared expression levels between the Müller cell-enriched and -depleted fractions from both analytical approaches. Based on averaged normalized abundances, a one way analysis of variance (ANOVA) and the ratios between fractions were calculated to determine which proteins were significantly different between the two cell fractions and visualized by volcano plots (supplemental Fig. S3). Heat maps generated from abundances of differentially expressed proteins (ANOVA, *p* < 0.05) by hierarchical clustering show clear separation between both fractions and illustrate close clustering of biological replicates (Fig. 3*A*, 3*B*). Next, we checked for the congruency of the rate of enrichment in the respective cell population for those proteins identified with both approaches. From 1246 proteins that met this criterion, for 94.5% of the proteins iST and cellular subfractionation yielded the same tendency of protein enrichment (either in Müller cells or retinal neurons) (Fig. 3*C*). For another 5.4% of proteins the enrichment did not reach significance in one of the approaches making the interpretation of the results difficult. Only for one protein (Atp1b1; 0.08% of all proteins) both approaches yielded a different enrichment pattern that reached significance in both instances.

In total we identified 497 proteins enriched in the Müller cell fraction (at *p* ≤ 0.05 and at least twofold more abundant) with 381 proteins derived only from the iST approach, 64 proteins only from the subcellular fractionation and 52 proteins identified with both methods (Fig. 3*E*). In the Müller cell-depleted neuronal fraction we identified a total of 607 proteins enriched (at *p* ≤ 0.05 and at least twofold more abundant) with 436 proteins derived only from the iST approach, 117 proteins only from the subcellular fractionation and 54 proteins identified with both methods (Fig. 3*D*).

##### Identification of Müller Cell-specific Molecular Functions

After Müller cell and neuronally enriched proteins were identified, the respective protein lists for the iST and subcellular fractionation approach were subjected to pathway enrichment analysis using Genomatix Generanker. We found highly differential activity patterns between the Müller cell-enriched and Müller cell-depleted neuronal fractions regarding enriched signal transduction mechanisms, molecular function of enriched proteins, their putative subcellular localization, the biological processes they are involved in, and the typical tissue identified on basis of the enriched protein expression pattern (Table I and II). Importantly, both sample preparation protocols yielded largely comparable results. In the Müller cell-depleted fraction pathways associated with light stimulus detection were strongest enriched—*e.g.* the most significantly enriched signal transduction pathway was “visual signal transduction in rods”, followed by the respective mechanisms in cones. Interestingly, most prominent molecular function of proteins enriched in retinal neurons was RNA binding and all kinds of RNA processing specifically enriched in the iST samples (Table I) in which there is no bias for membrane associated proteins like in the subcellular fractionation method. Other functions of neuronally enriched proteins included various mechanisms essential for energy derivation in the context of oxidative phosphorylation which is well in line with the immense need for energy especially of photoreceptor cells. For example, we found a significant enrichment of “NADH dehydrogenase, ATPase and hydrogen ion transmembrane transport activity” and looking at the enrichment at the level of cellular components the mitochondria play a central role (Table I, II, and supplemental Tables S3, S4).

*Vice versa*, pathway enrichment analysis clearly identified features of glial cells identifying Müller cells (apart from astrocytes) as likely cellular entity (Table I, II supplemental Tables S5, S6). Regarding dominant cellular functions of Müller cell proteins, focal adhesion kinase 1- , serum/glucocorticoid regulated kinase and tyrosine protein kinase src-mediated signaling pathways were top hits (Tables I and II). Calcium signaling appears to be a prevalent intracellular signaling mechanism in Müller cells because “protein kinase C (PKC), calcium ion binding and calmodulin binding” are in the list of top enriched molecular functions in the Müller cell fraction (Table I). Interestingly, anti-inflammatory (phospholipase inhibitors, annexins) and antioxidant proteins (numerous oxidoreductases and enzymes involved in glutathione metabolism) were strongly enriched in Müller cells. In contrast to the neuronal fraction, where intracellular membrane organelles (*e.g.* mitochondria, photoreceptors discs) were the dominant cellular components, cytoplasmic proteins, cell-cell-contact formation and cytoskeletal dynamics including vesicular transport and phagocytosis were impressively enriched in the Müller cell fraction (Tables I, II and supplemental Tables S5, S6).

##### Verification of Selected Müller Cell Enriched Genes Using qRT-PCR

**Fig. 4.**
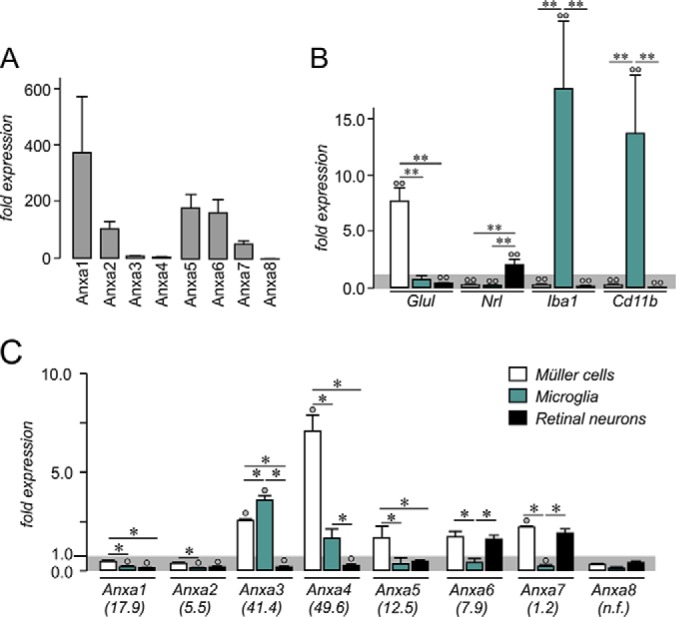
**Quantitative RT-PCR (qRT-PCR) validation of selected anti-inflammatory genes identified as enriched in Müller cells.** *A*, The general expression levels (mRNA) of the chosen anti-inflammatory genes in whole retinal extracts were determined to allow an estimation of the putative relevance of the respective gene in retinal physiology. Values represent the mean ± S.E. from three independent experiments. *B*, To obtain information about the annexin expression in microglia, we isolated this cell type in an additional enrichment step from the same retinal cell suspension from which subsequently also the Müller cells were isolated. Quantitative expression analysis using qRT-PCR confirms high purity of Müller and microglial fractions as the Müller cell specific glutamine synthetase (Glul) is highest expressed in the Müller cell fraction, whereas Nrl (photoreceptor marker) is primarily expressed in the neuronal fraction and the microglial markers Iba1 and CD11b are exclusively expressed in the CD11b-positive microglial fraction. Values are derived from four independent experiments for each analysis with retinae from two mice pooled per analysis. *C*, Expression analyses of annexin 1–8 at mRNA level in Müller cells compared with retinal neurons largely mirror the results obtained by quantitative proteomic analysis. Of note, the Müller cell-specific expression of all genes appears to be more pronounced at protein levels (for comparison the respective factor of enrichment taken from the proteomic analysis is given in the brackets below the gene symbol; annexin 8 (Anxa8) was not detected in our proteomic analyses, n.f., not found). Only Anxa3 showed highest expression levels in microglia, whereas most of the other annexins were primarily expressed in Müller cells (Anxa1, Anxa2, Anxa4, Anxa5). Values represent the mean ± S.E. from 5–6 independent experiments. For each experiment, cells were pooled from 4 retinae originating from 2 animals. *B, C,* **p < 0.01, *p < 0.05; significantly different expression level of the gene in the respective sample compared with whole retinal extracts: ○○*p* < 0.01, ○*p* < 0.05; Expression levels were normalized to the respective gene expression level detected in whole retinal extracts (*gray* shaded background). Anxa, annexin.

We validated our proteomic data with a second methodological approach choosing a protein family for which so far no expression in Müller cells has been described: the calcium-binding, anti-inflammatory annexins. First we determined the overall expression levels of all genes in whole retina preparations and found that annexin 1 and 2 are the genes most abundantly expressed (Fig. 4*A*). Because an expression of annexins is primarily described for microglia rather than macroglia (*e.g.* astrocytes) in the literature (27–29), we checked for their expression in acutely isolated microglia as well. Addition of a CD11b-microbead based positive selection for microglial to our Müller cell enrichment protocol yielded a very pure microglia fraction in addition to the Müller cell-enriched and Müller cell-depleted (neuronal) fraction (Fig. 4*B*). Except for annexin 3 and 8, all of the annexin family members were significantly lower expressed in microglia compared with the Müller cells at the mRNA level confirming the reliable output of our proteomic approach (Fig. 4*C*).

Next, we checked for annexin expression patterns in our Müller cell-enriched and -depleted samples and could largely confirm the results from quantitative mass spectrometry (Fig. 4*C*). Interestingly, in most cases there appears to be clear transcript-to-protein dosage linkage, meaning, if the protein was found to be highly enriched in Müller cells (*e.g.* annexin 3, 4, 5), the respective transcript was similarly strongly enriched compared with proteins with low enrichment in Müller cells for which also the transcript levels indicated a rather moderate enrichment (*e.g.* annexin 2, 7). For annexin 1 and 6 results from mass spectrometry and qRT-PCR differ, possibly indicating additional mechanisms modulating protein expression at other levels apart from mRNA levels.

##### Functional Validation of Protection Against Oxidative Stress From Retinal Müller Cells

**Fig. 5.**
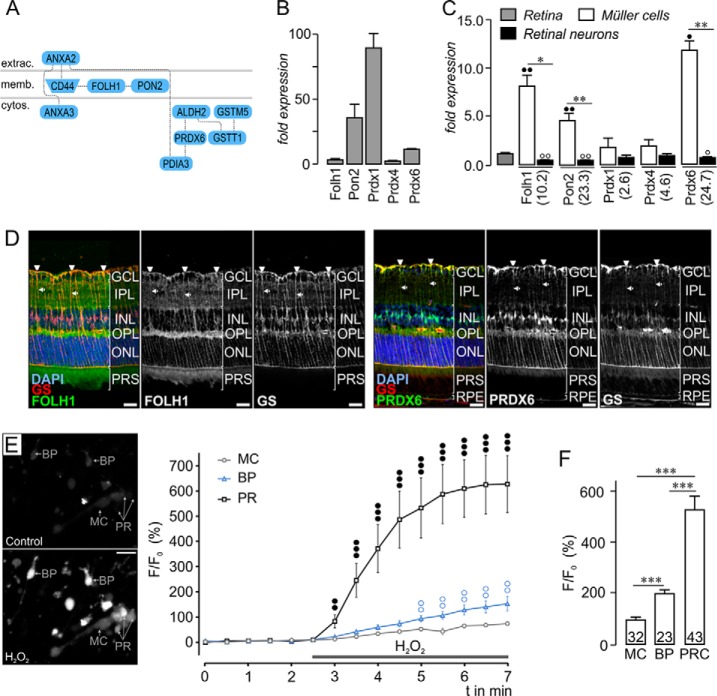
**Functional verification of the efficient protection of Müller cells against oxidative stress.** *A*, Exemplary protein network generated with proteins significantly enriched in Müller cells and with anti-oxidant activity revealed a significant accumulation of novel putative protectants against oxidative stress such as folate hydrolase 1 (FOLH1), paraoxonase 2 (PON2) and peroxiredoxin 6 (PRDX6) in Müller cells together with typical Müller cell markers (*e.g.* CD44) and already described protective Müller cell-specific enzymes such glutathione S-transferase T1 (GSTT1) and glutathione S-transferase mu 5 (GSTM5) in the Müller cell enriched fraction. Extrac., extracellular; memb., membrane; cyto., cytosol. *B*, Comparison of gene expression levels of selected antioxidant candidate genes detected by qRT-PCR performed on samples from whole retinal RNA extracts. *C*, Results from qRT-PCR experiments detecting transcript levels of selected genes with putative antioxidant activity in samples from whole retinal extracts (*gray* bar), enriched Müller cells (*white* bars) and the Müller cell-depleted neuronal fraction (*black* bars). Values represent the mean ± S.E. from four–five independent experiments. For each experiment, cells were pooled from four retinae originating from two animals. The values given in brackets below the gene symbol indicate the mean factor of enrichment of the respective protein as identified by the proteomic analysis. Significant difference between expression in Müller cell-enriched samples compared with Müller cell-depleted neuronal fractions: **p < 0.01, *p < 0.05; Significant difference between expression in Müller cell-enriched samples compared with whole retinal extracts: ••*p* < 0.01, •*p* < 0.05; Significant difference between expression in Müller cell-depleted samples compared with whole retinal extracts: ○○*p* < 0.01, ○*p* < 0.05. *D*, The subcellular localization of selected candidate genes in retinal sections was delineated by co-immunolabelings with the Müller cell marker glutamine synthetase (GS). PRDX6 is specifically expressed in Müller cells at presumably high levels and distributed throughout the whole cell body including their endfeet (open arrowheads) as well as their inner (arrows) and outer stem processes. Labeling for FOLH1 was less intense, but clearly confined to putative Müller cell structures such as their endfeet lining the inner retinal surface (open arrowheads) or their cell processes in the outer plexiform layer (OPL). Scale bars, 20 μm. GCL, ganglion cell layer; IPL, inner plexiform layer; INL, inner nuclear layer; ONL, outer nuclear layer; PRS, photoreceptor segments; RPE, retinal pigment epithelium. *E*, Characterization of reactive oxygen species (ROS) formation in response to H_2_O_2_ application in isolated retinal cell subtypes using CM-H2DCFDA. *Left*, representative images were taken from recording CM-H2DCFDA fluorescence detected in bipolar cells (BP), Müller cells (MC) and photoreceptors (PR) before and after 15 min of H_2_O_2_ (100 μm)exposure. Scale bars, 20 μm. *Right*, time course of alterations in the fluorescence intensity in the respective cell type (*n* = 10 each). Whiskers indicate S.E. values. Comparison of ROS levels in Müller cells *versus* photoreceptors: ••*p* < 0.01; •••*p* < 0.001. Comparison of ROS levels in Müller cells *versus* bipolar cells: ○○*p* < 0.01. *F*, In line with our expression data pointing to an excellent protection of Müller cells against oxidative stress, statistical analysis reveals the lowest levels of ROS formation in Müller cells that was significantly less pronounced compared with the response detected in bipolar cells and photoreceptor cells - the latter being most susceptible to H_2_O_2_-induced ROS formation. Numbers given in the bars indicate the numbers of measured cells. Values represent the mean ± S.E. Experiments were repeated for cell isolations from three different animals. ***p < 0.001.

Another interesting protein family indicated to be significantly enriched in Müller cells by pathway enrichment analysis (Table I) contains proteins with antioxidant activity. Fig. 5*A* gives an overview about the putative interaction of these proteins together with known Müller cell markers such as CD44 or glutathione-S-transferase. Again, we first determined the general expression level of selected putatively antioxidative proteins in the retina and detected highest expression levels for paraoxonase 2 (Pon2), peroxiredoxin (Prdx) 1, 4, and 6 (Fig. 5*B*). Expression analysis on the isolated Müller cell subpopulation confirmed a significant enrichment of all the selected genes except Prdx1 where the tendency of a Müller cell-specific expression did not reach significance at mRNA level (Fig. 5*C*). Immunolabeling on retinal sections further illustrated the subcellular distribution of the two clearly Müller cell-specific proteins—folate hydrolase 1 (FOLH1) and PRDX6. Although the labeling of FOLH1 was rather faint but clearly located in Müller cell structures such as their endfeet lining the inner retinal surface, PRDX6 could be massively detected throughout the whole Müller cell body (Fig. 5*D*). Accordingly, PRDX6 could serve as novel Müller cell marker. To further verify specificity of PRDX6 expression in Müller cells, semiquantitative immunofluorescence experiments on dissociated single retinal cell types were performed that clearly confirm the data from mass spectrometry, qRT-PCR and immunolabelings of retinal slices (supplemental Fig. S4*A*). Moreover, Müller cell-specific PRDX6 protein expression was confirmed by Western blot (supplemental Fig. S4*B*).

Given this impressive Müller cell-specific expression of PRDX6 (and the moderate enrichment of Prdx1 and Prdx4) - an enzyme family which has been demonstrated to be involved in detoxification of H_2_O_2_ (30, 31)—we went on to check whether Müller cells are more resistant to H_2_O_2_-induced ROS formation. To this end, we prepared retinal cell suspensions from acutely isolated retinae, the cells were loaded with the ROS-sensitive dye CM-H2DCFDA and were then exposed to H_2_O_2_. Indeed, we found photoreceptors to be most sensitive to H_2_O_2_-induced ROS formation, whereas Müller cells only showed a very moderate increase in CM-H2DCFDA fluorescence intensity after 15 min of exposure to H_2_O_2_ (Fig. 5*E*, 5*F*). We also included analysis of ROS formation in bipolar cells that can easily be identified on basis of their distinct morphology and because they are closely associated with Müller cells in the inner nuclear layer *in vivo*. A less pronounced rise in ROS levels upon H_2_O_2_-treatment was observed in bipolar cells compared with photoreceptors, yet significantly more ROS were detected in bipolar cells than in Müller glia (Fig. 5*E*, 5*F*).

In sum, we confirmed the results from quantitative mass spectrometry by qRT-PCR experiments, immunolabeling and, importantly, also on the cell functional level.

## DISCUSSION

Protocols to isolate glial cells from brain or retina are commonly confined to the use of tissues derived from postnatal animals at maximal 1 week of age (32, 33). This brings the pitfall of an expression analysis done on immature glial cells (33,34). For example Müller cells, the major macroglia of the retina, do not start to differentiate into their adult-like phenotype until the end of the first postnatal week (4, 35). We present here a novel approach to isolate large, fragile and arborized glial cells from the adult retina yielding a highly enriched cell population that retains *in situ* morphology and is thus well suited for proteomic analyses. With high efficiency we recover >65% of the total Müller cell population per retina which enables the application of complementary proteomic strategies to maximize identification rates while still keeping necessary cell counts at a minimum. With this approach we could for the first time deeply cover the Müller cell proteome, directly compare expression levels to retinal neurons and thus uncover proteins enriched or exclusively expressed in this important glial population in the retina.

### 

#### 

##### Novel MACS-based Müller Cell Enrichment Protocol

Cell type-specific analysis of Müller cell gene expression was previously confined to studies performed on cell lines (*e.g.* MIO-M1 (36)) or primary Müller cell cultures (15, 16, 37) owing to challenges of isolating intact native Müller cells from retinal tissues. However, profiling of primary Müller cells even after short times in culture does not result in data sets resembling the *in vivo* proteome, because the cells dedifferentiate and profoundly change their proteome soon after being taken into culture (15). Acute isolation of intact Müller cells is hampered by their huge cell size (up to 200 μm length in mouse retina) in combination with their close physical interaction to retinal neurons, microglia and blood vessels via membrane specializations partially enwrapping the neighboring cells and structures. Consequently, enrichment by popular tools such as FACS will result in partially destroyed and lost subcellular structures, which is a severe drawback especially for proteomic profiling. The few previous attempts to acutely enrich Müller cells for expression profiling included cell isolation by picking single cells under the microscope resulting in low sample yield (but high purity) unsuitable for proteomic approaches (13, 38), FACS sorting on basis of enhanced green fluorescent protein expression (14), which is reliant on the respective transgenic mouse strain and disrupts the Müller cell morphology tearing apart major cell processes and a MACS-based approach using an anti-glutamate-aspartate-transporter (GLAST)-antibody and postnatal retinal tissue resulted in a maximal enrichment of Müller cells of 30% (32). We present here a novel MACS-based approach of Müller cell enrichment using integrin beta-1 (Itgb1) as surface marker that yields very high Müller cell purity (up to 90%, Fig. 1*B*) while maintaining mostly intact morphology (Fig. 1*A*) from adult murine retina independent from the genetic background and with sufficient sample output (∼150 000 Müller cells/retina). Given the huge cell size of Müller glia that results in a comparatively high protein yield per cell and the high purity of those Müller cell-enriched fractions, the proteome identified from these cell preparations presumably represents mainly the Müller cell proteome. However, putative minor contamination with other cell populations or disrupted cell processes still attached to the Müller cell are likely to occur *e.g.* from microglia cells (∼3000–4000 per murine retina, own unpublished data and (39)) and cells of the retinal vasculature (pericytes, endothelial cells) because these cell types interact closely with Müller glia. However, histological analysis of enriched cell populations demonstrated that the main contaminating cells in Müller cell-enriched fractions are photoreceptors, which also closely interact *in vivo*. Because highly abundant photoreceptor-specific proteins such as phosphodiesterase 6 subunits, S-antigen, cyclic nucleotide gated channel subunits and rhodopsin are efficiently depleted from the Müller cell-enriched fractions (between four- and 20-fold depletion, see supplemental Tables S1, S2), we conclude that protein abundances from photoreceptors are reasonably low and those from other contaminants are likely below detection thresholds.

With this approach we achieved for the first time to effectively enrich adult Müller cells which are morphologically intact at high purity and with sufficient yields for proteomic profiling.

##### The Müller Cell Proteome

Although the novel isolation protocol yields highly enriched Müller cell preparations, application of a label-free quantitative LC-MSMS approach (16, 23, 40) enabled us to directly compare Müller cell-enriched preparations to their counterpart Müller cell-depleted fractions, which consist of more than 50 different cell types (41) and are almost purely retinal neurons (>98% purity, Fig. 1B). Housekeeping proteins, which are present at similar amounts in all cell types, improve the robustness of the quantitative comparison, as has been recently also proposed for label-free quantification of purified protein complexes, where contaminating proteins serve this purpose (42). We aimed to maximize the total number of identified and quantified proteins by applying two conceptually different strategies of sample preparations, both aiming at minimizing necessary sample input. The recently described lossless digestion of low sample amounts iST combined with biochemical fractionation of the resulting peptide pool (21) was efficient enough to result in 3077 protein IDs from only 200,000 Müller cells and ∼500,000 retinal neurons (both yielding less than 10 μg total protein input). Although cell number restrictions critically affect identifications rates, it has been often demonstrated that complementary analytical strategies are most effective in increasing coverage in proteomic experiments. Therefore we downscaled a subcellular fractionation approach which we recently used for cell surface protein profiling in MACS-isolated human CD4 T-cells (19). Although using as many as 8 million naïve T-cells for biotinylation resulted in identification of 173 putative cell surface proteins, using between 0.5 and 0.8 million Müller cells and ∼1.5 million retinal neurons in this study resulted in identification of 111 putative cell surface proteins in the biotinylated fraction. Our data set also compares very well with another recent study using a different cell surface labeling approach on a total of 72 cell lines and cell types (Cell Surface Protein Atlas; (43)) where extraordinary high input amounts (1 × 10^8^ suspension cells or 200 mg to 1g of tissue or 5 × 15 cm dishes at 80% confluency) yielded an average identification rate of 284 putative cell surface proteins per cell type.

The proportion of overlapping proteins identified in our enriched cell populations by both analytical strategies is rather high with 80.2% of the proteins identified in the subcellular fractionation approach also detected with the iST approach indicating a reasonable analytical depth (Fig. 2*A*). However, because the iST approach identifies significantly more proteins and the overlapping identifications to the subcellular fractionation is only 43%, we assume that we do not yet have full coverage of the proteome. Further optimizations as well as more sensitive mass spectrometric equipment will enable improvement of analytical depth even further in future data sets.

##### Pathway Enrichment Analysis Confirms Strength of the Proteomic Approach

As a result from the combination of iST and subcellular fractionation approach, we identified a set of 497 proteins more abundantly expressed in Müller cells and 607 proteins more abundantly in retinal neurons (Fig. 3*D*, 3*E*). We subjected the lists of proteins significantly enriched in the Müller cell-enriched or -depleted fractions as identified either by iST or subcellular fractionation to GeneRanker analysis addressing the question whether the two methods of protein processing yield information about different protein classes (*e.g.* membrane-associated *versus* cytoplasmic proteins). The enrichment analyses clearly identified the Müller cell fractions to be of neuroglial/astrocytic origin even identifying them as Müller cells (Tables I and II) irrespective of the applied protocol for sample preparation. Similarly, pathway enrichment analysis assigned a rod photoreceptor phenotype at highest significance level to the neuronal fraction with cone photoreceptor phenotype being identified at a magnitudes lower significance level (Tables I and II). This is well in line with the fact that rod photoreceptors make up 78.5% of all cells in the retina, whereas the murine retina comprises only 2.2% cones (12). It is worth mentioning, that although photoreceptors and other retinal neurons make up ∼10% contaminating cells in the Müller cell enriched fractions, no phototransduction-specific pathways were overrepresented in the GeneRanker analyses, substantiating the specificity of the approach.

Pathway enrichment analyses also gave insight into cell type-specific signal transduction mechanisms, prominent biological processes and overrepresented cell compartments where these processes occur. Of note, at this level of analysis slightly different results were obtained from the iST compared with the subcellular fractionation approach with the latter being more efficient in identifying processes occurring at cell membranes. Enriched signaling pathways or cell functions identified with the iST protocol mainly allocate to intracellular compartments or intracellular downstream signaling events. Accordingly, proteins associated with cytoskeletal functions as well as those mediating neurotransmitter recycling dominate in Müller cells (Table I). In addition and as expected from various physiological studies on vital retinal preparations, signal transduction involving calcium as second messenger dominates in Müller cells as cellular functions such as their volume regulation, glio-vascular coupling, mechanosensitivity and glia-to-neurons in the context of the retinal light response have been demonstrated to be mediated by Müller glial calcium responses (35, 44–47).

*Vice versa*, analysis of Müller cell-specific proteins identified by subcellular fractionation result in overrepresentation of plasma membrane compartment reflecting the intimate cross-talk of the cell with the extracellular compartment. Establishment of cell-cell-interactions involving focal adhesion kinase signaling and cytoskeletal remodeling is of key relevance for Müller cells (Table II) pointing to their essential role in structural organization of the retinal morphology (48). An enriched signal transduction pathway was that mediated by tyrosine protein kinase src (Table II). Src kinases are an integral part of integrin-mediated signaling mechanisms modulating cytoskeletal reorganization in response to external stimuli and interact with various growth factor receptors (49). Strong expression of proteins associated with src kinase function corroborates the central role of Müller cells interacting with their extracellular surrounding, but is also in line with our recent findings that numerous growth factors (VEGF, erythropoietin, NGF) that exert beneficial effects on Müller cell physiology (*e.g.* their cell volume regulation) act via stimulation of receptor tyrosine kinase activity and putative downstream involvement of src kinase (50–52).

Enrichment analysis of Müller cell-specific proteins identified by subcellular fractionation also indicated that Müller cells are well-equipped for transmembrane transport of neurotransmitters which mirrors their key role in clearing neurotransmitters such as glutamate and γ-amino butyric acid (GABA) from the synaptic cleft thereby terminating synaptic transmission (53–55). Finally, intracellular transport mechanisms, vesicle formation and packaging are biological processes significantly enriched in Müller cells (Tables I and II) supporting the hypothesis of Müller cells generating a neuroprotective environment in the retinal tissue by production and secretion of neurotrophic factors (37, 56–59).

In contrast, the two most prevalent biological processes in Müller-cell depleted samples, containing all other retinal cell types with mostly retinal neurons (41), were those of RNA processing and cellular respiration (Tables I and II) (apart from the expected enrichment of pathways associated with phototransduction). The first finding indicates an immense transcriptional and very likely also post-transcriptional and translational activity in retinal neurons probably reflecting the constant need to renew photoreceptor outer segment components. Photoreceptors shed ∼7% of their outer segment mass per day which contains the photoreceptors disks densely packed with proteins involved in phototransduction (60, 61). The enormous enrichment of components essential for the oxidative energy generation reflect the fact that retinal neurons and especially photoreceptors have an extraordinary energy demand (62) which makes the retina the tissue with the highest oxygen and glucose consumption per weight unit. Because rods and cones are characterized by a high degree of compartmentalization, with all biochemical reactions involved in generating light responses being confined to their outer segment organelle and energy generation taking place in mitochondria residing in the inner segment, analysis regarding most relevant cellular components identified many membranous components such as mitochondria, the cilium membrane and the photoreceptor outer segments. *Vice versa*, proteins present in the cytoplasm, components of the cytoskeleton and relevant for vesicle formation and trafficking were strongly enriched in Müller cells.

In line with the fact that phototransduction and oxidative phosphorylation occur in membrane compartments both pathways were largely overrepresented in samples derived from subcellular fractionation (Table II), whereas the intense neuronal RNA processing activity was more clearly identified in the iST sample preparations (Table I). These findings together with the results for the Müller cell fraction described before points to the fact that subcellular fractionation allows better analysis of membrane-associated proteins and that the iST protocol yields more unbiased information of protein expression modulating all other (intra-) cellular functions.

##### Results From Proteomic Analysis Could be Verified at mRNA and Cell Functional Level

Using qRT-PCR to determine the gene expression at mRNA level, we sought to validate the expression of a selected set of proteins that had been identified to be enriched in Müller cells with mass spectrometry. Although it is well described in numerous studies that levels of mRNA do not necessarily reflect protein abundances, significant correlation has been observed in a recent large-scale study (63) justifying this validation approach. In a first set of experiments we chose the annexin gene family of calcium-dependent membrane-binding proteins for validation. Little can be found in the literature about the expression of this protein class in glial cell types and even less about their expression in the retina. Mass spectrometry measurements indicated a significantly enriched expression of Anxa1 to Anxa6 in Müller cells and we could confirm the respective expression pattern via qRT-PCR at mRNA level except for Anxa6 (Fig. 4*C*). Strongest enrichment was detected for Anxa1, Anxa3 and Anxa4. In comparison, Eberhard *et al.* (64) report the expression of Anxa1, Anxa2 and Anxa4 in reactive hippocampal astrocytes from the diseased human brain, but only detected low expression levels in normal astrocytes. Studies performed with whole retinal sample collections describe a contribution of Anxa2 and Anxa3 in neovascularization neonatal models of oxygen-induced retinopathy where they were significantly up-regulated (65, 66). Thus, it would be highly interesting to analyze the Müller glial expression of respective annexins in similar disease models to elucidate the role of glial annexins in neoangiogenesis. Apart from this, Anxa1 (also known as lipocortin-1) was typically found to be expressed in microglia (27, 67) exerting an anti-inflammatory effect. Here we compared mRNA expression levels of Anxa1 and all other annexin family members in retinal microglia with that in Müller cells and found a higher basal expression level of all annexins in Müller cells, except for Anxa3 for which Müller cell expression equals that in microglia. Given the enriched presence of proteins related to extracellular vesicular exosomes in Müller cells (Table 1) and the known localization of Anxa1 to exosomal vesicles (68–70), this may imply that Müller cell expression and presumably secretion of Anxa1 (and most likely other annexin family members) could preserve an anti-inflammatory environment in the retina thereby probably polarizing the microglial response toward a protective phenotype *e.g.* via stimulation of the phagocytic removal of apoptotic neurons while preventing the production of pro inflammatory mediators (27, 71).

Interestingly, many of the Müller cell-enriched proteins identified with our approach are known to be involved in oxidative defense mechanisms (*e.g.* Gstm5, Gstt1, Pon2, Prdx1, Prdx6) (30, 31, 72–74). Of note, a reduced transcript level of GSTM5 and GSTT1 in human retinae and retinal pigment epithelium (RPE) owing to promoter hypermethylation have been associated with an increased risk to develop age-related macular degeneration - a retinal disease associated with oxidative stress induced RPE and neuronal degeneration (75). Moreover, a null-allele of GSTT1 has been associated with an enhanced risk to develop diabetic retinopathy in type 2 diabetic patients (76).

To validate the results from the proteomic approach and to verify the antioxidant potential of Müller glia, we first confirmed the respective expression patterns of all selected antioxidant candidate genes that so far have not been described to be expressed in Müller cells (Pon2, Prdx1, Prdx4, Prdx6) and Folh1, an enzyme cleaving N-acetyl-l-aspartyl-l-glutamate thereby generating free glutamate that can be used for the generation of the antioxidant glutathione in Müller cells (77) using qRT-PCR (Fig. 5*C*) and partially also by immunolabeling. Especially the labeling for Prdx6 clearly and very comprehensively delineated the Müller cell morphology (Fig. 5*D*). Accordingly, Prdx6 can be considered as a potent novel marker for Müller cells in the murine retina. Of note, Prdx6 just recently has been identified as an important player in diabetes pathology as knockout of Prdx6 led to impaired insulin production and reduced muscular glucose up-take in mice (78). Moreover, neuroprotective effects of high Prdx6 levels have been demonstrated recently (79). Given its strong enrichment in Müller cells and described alterations of Müller cell functions in diabetic retina that are partially because of diabetes-induced oxidative stress (80–82), investigations on the functional role of Prdx6 in normal Müller cells and, importantly, in Müller cells of the diabetic retina would be of highest interest to elucidate whether altered expression levels are associated with the development of diabetic retinopathy.

To confirm that Müller cells possess a superior anti-oxidative stress response we compared their ROS formation upon exposure to H_2_O_2_ to retinal neurons (Fig. 5*E*, 5*F*). Indeed, we found that Müller cells are highly resistant to oxidative stress especially when comparing their response to that of photoreceptor cells that showed an almost six times higher production of ROS (Fig. 5*F*). Besides other antioxidant enzymes such as Gstm5 and Gstt1, Prdx6 might promote a major part of this Müller cell protection as its efficient detoxification of H_2_O_2_ has been described byothers (30, 31). Verification of Prdx6 involvement has to be confirmed further in future experiments on *e.g.* Müller cells derived from Prdx6-deficient mice.

## CONCLUSION

Our novel approach to isolate intact glial cells from adult retina in combination with proteomic profiling enabled the identification of novel Müller glia specific proteins. This provides the basis to discover novel glial specializations and reveals novel Müller glia markers that will be instrumental for future exploration and understanding of gliosis in diseases.

## Supplementary Material

Supplemental Data

## Footnotes

^1^ The abbreviations used are:

FACS: fluorescence-activated cell sorting
2D: two-dimensional
ABC: ammoniumbicarbonate
ACN: acetonitrile
ANOVA: analysis of variance
Anxa: Annexin
CM-H2DCFDA: 5-(and-6)-chloromethyl-2′,7′-dichlorodihydrofluorescein diacetate, acetyl ester, general oxidative stress indicator
CRALBP: glutamine synthetase and cellular retinaldehyde binding protein
DMSO: dimethyl sulfoxide
DAPI: 4′,6-diamidino-2-phenylindole
ECS: extracellular solution
GABA: glutamate and γ-amino butyric acid
GdmCl: guanidinium chloride
GePS: Genomatix Pathway Sytem
GLAST: glutamate/aspartate transporter
Glul: glutamine synthetase (mRNA)
GS: glutamine synthetase (protein)
GSTM5: glutathione S-transferase mu 5
GSTT1: glutathione S-transferase T1
FASP: filter aided sample preparation
FDR: false discovery rate
FOLH1: folate hydrolase 1
Iba1: ionized calcium binding adapter molecule 1
iST: in-StageTip protein sample preparation
Itgb1: integrin beta-1
LC-MSMS: high resolution mass spectrometry coupled to liquid chromatography
MACS: magnetic-activated cell sorting
mgf: Mascot generic files
OPL: outer plexiform layer
PBS: phosphate-buffered saline
PKCα: protein kinase C alpha
Pon2: paraoxonase 2
Prdx: peroxiredoxin
PVDF: polyvinylidenfluoride
qRT-PCR: quantitative real time polymerase chain reaction
ROS: reactive oxygen species
RPE: retinal pigment epithelium
SEM: standard error of the mean
TBS: tris-buffered saline
TCEP: tris(2-carboxyethyl)phosphine
TFA: trifluoroacetic acid.
