# Supplementary material for: The Proteome of Native Adult Müller Glial Cells From Murine Retina
Source: Mol Cell Proteomics. 2015 Aug 31;15(2):462–80. doi: 10.1074/mcp.M115.052183 (PMC4739667; doi:10.1074/mcp.M115.052183)
Supplement: Supplemental Data [file supp_15_2_462__index.html]

The proteome of native adult Muller glial cells from murine retina — The Proteome of Native Adult Müller Glial Cells From Murine Retina — The Müller Cell Proteome — Supplemental Data 

# The Proteome of Native Adult Müller Glial Cells From Murine Retina

## Supplemental Data

- supplemental table 1 - Identified proteins and peptides from iST approach
- supplemental table 2 - Identified proteins and peptides from subcellular fractionation approach
- supplemental table 3 - Pathway enrichment analyses of neuronal fraction from iST approach
- supplemental table 4 - Pathway enrichment analyses of neuronal fraction from subcellular fractionation approach
- supplemental table 5 - Pathway enrichment analyses of Muller cell fraction from iST approach
- supplemental table 6 - Pathway enrichment analyses of Muller cell fraction from subcellular fractionation approach
- supplemental table 7 - iST elution buffer composition
- supplemental table 8 - sequence coverages of all identified proteins
- supplemental figures 1 to 4 - supplemental figures 1 to 4
