# Supplementary material for: The Proteome of Native Adult Müller Glial Cells From Murine Retina
Source: Mol Cell Proteomics. 2015 Aug 31;15(2):462–80. doi: 10.1074/mcp.M115.052183 (PMC4739667; doi:10.1074/mcp.M115.052183)
Supplement: Supplemental Data [file supp_M115.052183_mcp.M115.052183-9.pdf]

Supplemental Figures

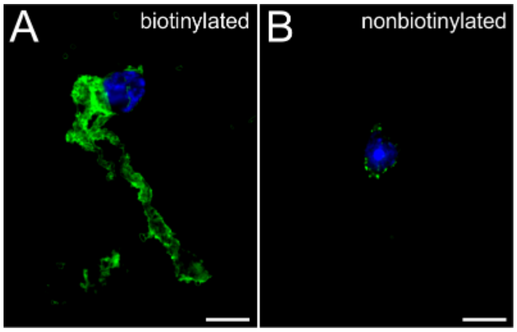

Suppl. Fig. 1. Surface proteins of vital cells were biotinylated to allow discrimination from cytosolic proteins. Demonstrating successful biotinylation, nonpermeabilised cells were incubated with Alexa488-coupled streptavidin (*green*). No surface labelling was observed for nonbiotinylated cells. Scale bars, 10 µm.

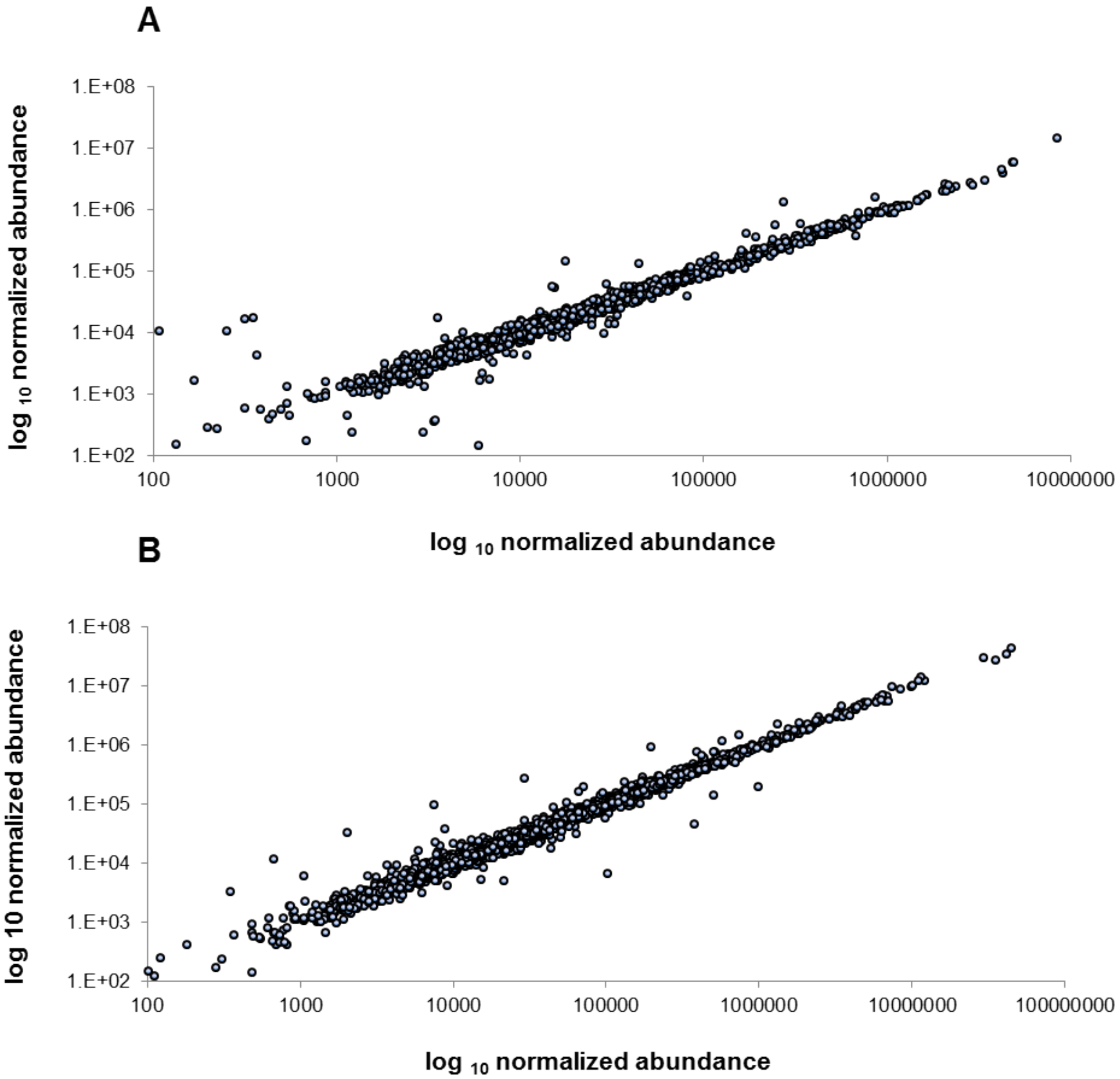

Suppl. Fig. 2. Technical reproducibility of label-free quantification of RMG using subcellular fractionation (A) and iST sample preparation (B). Technical reproducibility of the LC-MSMS measurement was assessed in 2 replicates. Plotting $\log_{10}$ normalized abundance values for each protein in replicate 1 (x-axis) against replicate 2 (y-axis) reveals high correlation of quantification for both approaches (iST, r2 0.98; subc. fract., r2 0.93). The mean coefficient of variation across all quantified proteins was 8.44 % in the iST approach (n = 3077) and 9.58 % in the subcellular fractionation approach (n = 1649).

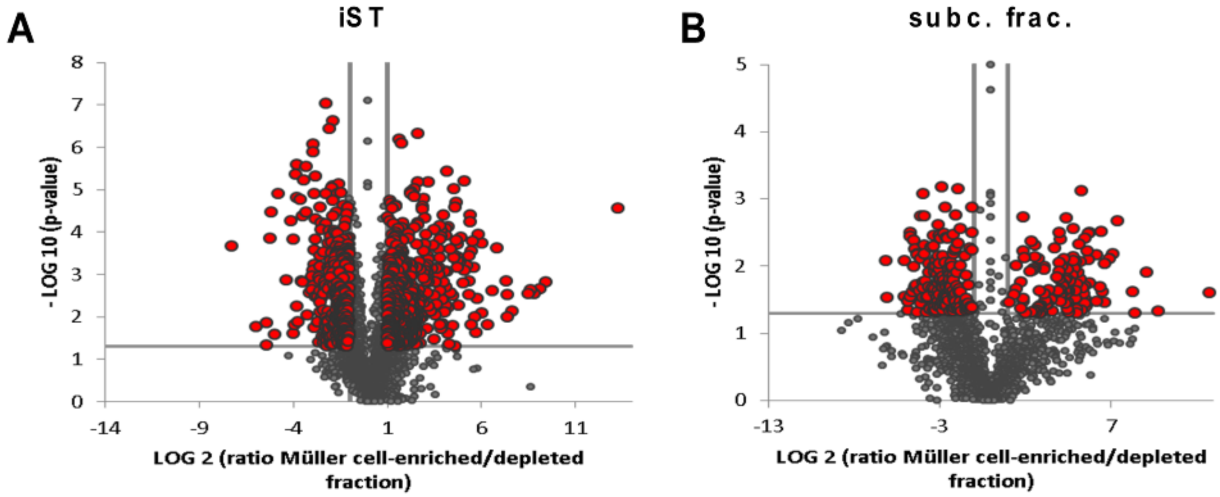

Suppl. Fig. 3. Volcano plots showing *P* values (-log10) versus protein ratio of Müller cell-enriched/depleted fractions (log2) of all 3077 proteins (iST approach, A) and 1649 proteins (subcellular fractionation approach, B). Red dots on right sides represent Müller cell-enriched proteins; red dots on left sides represent neuronal cell-enriched proteins; grey, not significantly changed upon between cell fractions; (ANOVA with $P < 0.05$)

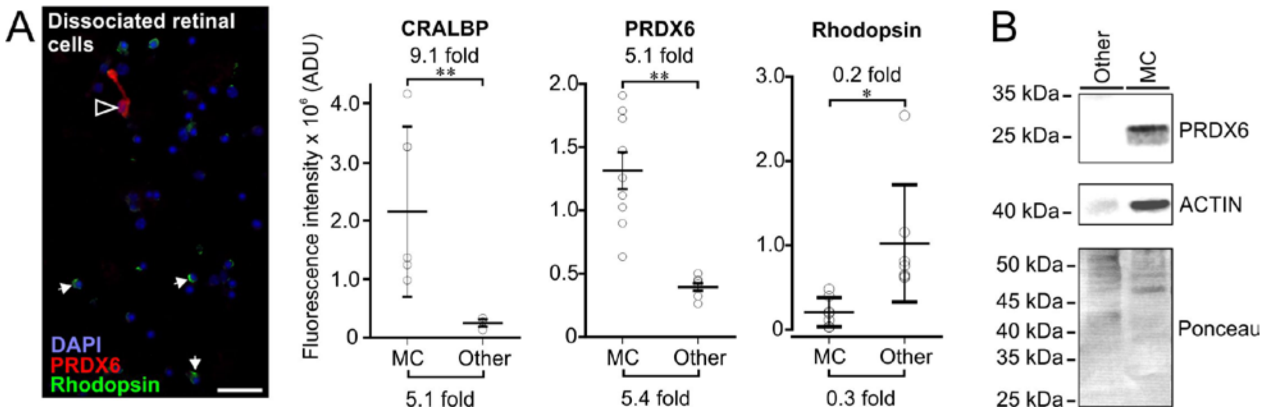

Suppl. Fig. 4 Validation of Müller cell-specific expression of Prdx6. (A) Left, Retinal tissue was dissociated, fixed and labeled for PRDX6 and rhodopsin or CRALBP to detect fluorescence levels from single cells. A PRDX6 positive Müller cells is marked by the open arrowhead, rhodopsin positive photoreceptor cell bodies by arrows. Owing to the isolation procedure photoreceptors loose the major part of the outer and inner segment. Scale bar, 50 µm. Right, the mean fluorescence intensity over the area of a cell was measured as semi-quantitative indicator of protein expression levels. Expression levels were compared between Müller cells (MC) and non-Müller cells (other). Values are given as mean ± SEM. Symbols represent mean values of an individual scan in which values from all cells present were summoned up. Samples were derived from 3 wild type mice. Numbers underneath the bracket gives the enrichment score of the respective protein determined by mass spectrometry. (B) Müller cells were enriched by MACS. Equal amount of total protein derived from the Müller cell-enriched and Müller cell-depleted cell fraction was subjected to Western blot analysis demonstrating the exclusive expression of Prdx6 in the Müller cell fraction. Actin, usually applied as house keeper, is primarily expressed by Müller glia. Owing to lack of suitable loading controls, Ponceau staining was performed to demonstrate loading of equal amounts of protein.
